# Rapid Recovery of Damaged Ecosystems

**DOI:** 10.1371/journal.pone.0005653

**Published:** 2009-05-27

**Authors:** Holly P. Jones, Oswald J. Schmitz

**Affiliations:** School of Forestry and Environmental Studies, Yale University, New Haven, Connecticut, United States of America; Northeastern University, United States of America

## Abstract

**Background:**

Recent reports on the state of the global environment provide evidence that humankind is inflicting great damage to the very ecosystems that support human livelihoods. The reports further predict that ecosystems will take centuries to recover from damages if they recover at all. Accordingly, there is despair that we are passing on a legacy of irreparable damage to future generations which is entirely inconsistent with principles of sustainability.

**Methodology/Principal Findings:**

We tested the prediction of irreparable harm using a synthesis of recovery times compiled from 240 independent studies reported in the scientific literature. We provide startling evidence that most ecosystems globally can, given human will, recover from very major perturbations on timescales of decades to half-centuries.

**Significance/Conclusions:**

Accordingly, we find much hope that humankind can transition to more sustainable use of ecosystems.

## Introduction

Humankind is heavily exploiting ecosystems to meet rising demands for resources and environmental services [1], [2], [3], [4]. An inevitable consequence of this impact is that biotic and biophysical conditions of ecosystems become degraded from overuse or from accidents [1], [2], [3], [4], [5]. Competing demands for finite space and finite ecosystem services [2], [5] means ultimately there will be limited if any recourse to abandon degraded areas and shift exploitation to non-degraded ones [2], [3]. Conservation efforts must therefore turn toward restoration of degraded environments in order to create the portfolio of future opportunities that balance environmental protection against providing environmental services for a burgeoning human population [1], [2], [3], [4], [6]. This necessarily begs the questions: is there any hope that ecosystems can recover from the perturbations they face [1]; and if so, how long will recovery take [1], [2], [3]?

In theory, ecosystems could recover gradually from perturbations at a rate proportional to the degree to which the perturbation is abated [7], [8], [9]. It is speculated nonetheless that such recovery will take centuries if not millennia given the scales of current human impact [1], [2], [3]. Alternatively, ecosystems could reach critical thresholds and entrain into alternative states thereby precluding recovery [10], [11], [12]. There is much uncertainty about which alternative is likely for a broad range of ecosystems [3]. Yet knowing these likelihoods is central to sustainable use of ecosystems [12], [13], [14]. We address this uncertainty by synthesizing 240 independent published studies of ecosystem recovery (Table S1).

## Methods

Our data set was derived from peer-reviewed studies that examined large, human-scale ecosystem (vs. small scale experimental) recovery following the cessation of a perturbation (Table S1). We conducted a search of the primary literature using Web of Science for the years 1910–2008 inclusive. We used the perturbation-type keywords agriculture, deforestation, eutrophication, hurricane, cyclone, invasive species, logging, oil spill, power plant, and trawling. To focus on recovery, we searched on the concatenated string of the following words: perturbation type AND resilience AND recovery. We excluded studies that focused on single species recovery. Studies included both experimental and natural perturbations and both passive and active recovery projects. For multiple studies that looked at the same perturbation, we used the most recent study. For those studies published in the same year, we selected the report that provided the greatest amount of empirical data.

We cross-compared our database with the threshold database provided by the Resilience Alliance (www.resalliance.org) and found that 236 of our cases did not overlap with the cases reported in the threshold database.

We grouped the data into broad categories of ecosystem types. Terrestrial ecosystems include old field, grassland, prairie, and scrub habitats. Forest systems include tropical and boreal forests. Freshwater systems include lakes, streams, and rivers. Brackish systems include marshes, wetlands, and swamps. Lastly, marine systems include coastal, benthic, pelagic, and lagoon habitat.

Most studies measured multiple response variables. We separated each response variable into one of three categories: ecosystem function, animal community, or plant community. Ecosystem variables included nutrient cycling, decomposition rates, and abiotic measurements. Animal and plant community variables included estimates of density, diversity, evenness, and species composition.

We quantified recovery of each of the variables in terms of the time it took for the variables to return to their pre-perturbation state. We used the recovery time reported by the authors for the particular study. We used the median recovery value whenever studies reported a range of recovery times. Each ecosystem or community variable was designated as recovered, headed towards recovery or not recovered. To ensure our analysis was unbiased, we excluded those variables that were headed towards recovery. When those variables were included into the recovered variables category (a more optimistic viewpoint), we found no changes to any of the conclusions in our results.

For each study, we quantified the time taken for a system to recover to a pre-disturbance state. We relied on the authors' own expert judgment, as declared in their studies, of whether or not their system had recovered. Individual studies typically reported recovery for more than one variable so we evaluated whether or not there were trends in the number and kinds of variables that either recovered or did not recover among ecosystem and perturbation types. We also assessed whether recovery time was related to the degree to which the system deviated from its initial conditions (perturbation magnitude).

**Figure 1:**
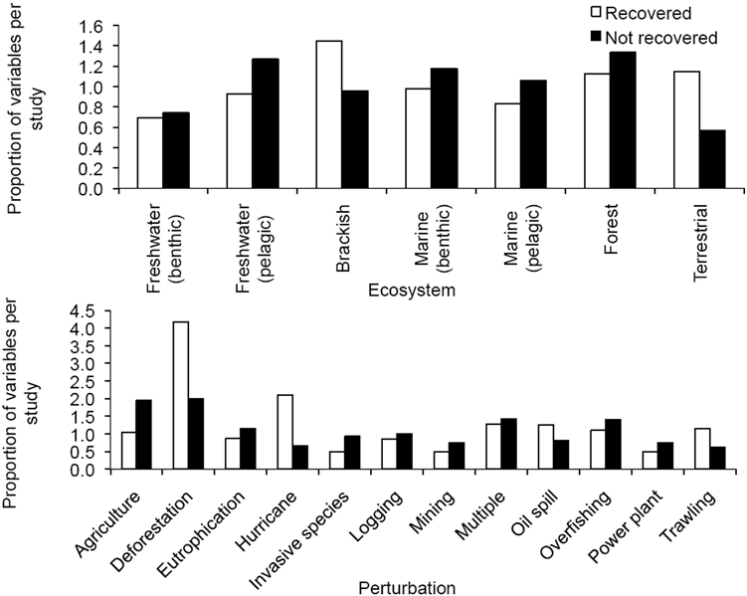
Proportion of variables per study that had recovered (white) versus variables that had not recovered (black) separated by ecosystem type (top) and perturbation type (bottom). We scaled the studies on a per study basis to avoid biasing our results toward ecosystems or perturbation types with higher representation (see text). Proportions are greater than one as a result of single studies having more than one response variable. Higher proportions indicate a higher incidence of recovery (white) or non-recovery (black). We found no significant differences between any of the paired variables, indicating an equal likelihood of recovery or not for all variables. These are conservative estimates of recovery likelihood as we excluded any variables that were headed towards recovery but had not yet fully recovered.

Individual studies typically measured more than one ecosystem and community variable. The challenge in assessing recovery of variables is to control for pseudoreplication due to multiple variables in a single study. One approach is to calculate the proportion of variables within a study that recovered or not and then calculate the average proportion of recovery or not across all studies. However, this approach gives equal weighting to studies with widely varying numbers of variables leading to an elevated contribution of studies with few variables to the overall conclusion about recovery. To overcome this potential bias, we calculated an index that considered the total number of variables that recovered or not by category (ecosystem or perturbation type) rather than by study. We then normalized the number of variables to the number of studies conducted in each category thereby eliminating pseudoreplication. Thus, for each ecosystem or perturbation type, we summed the number of recovered and non-recovered variables, respectively, using the formulae,
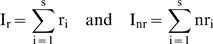
where r_i_ and nr_i_ are respectively the number of recovered and non-recovered variables in study i and s is the total number of studies for a given ecosystem or perturbation type. We scaled our measure by s, giving, effectively, a per study measure of recovery and non-recovery in order to make an unbiased comparison among ecosystems and perturbations for which there are different numbers of studies. The number of variables that recovered or did not recover per study was the same among ecosystem and perturbation types (t-test, p>0.06; Fig. 1).

**Figure 2:**
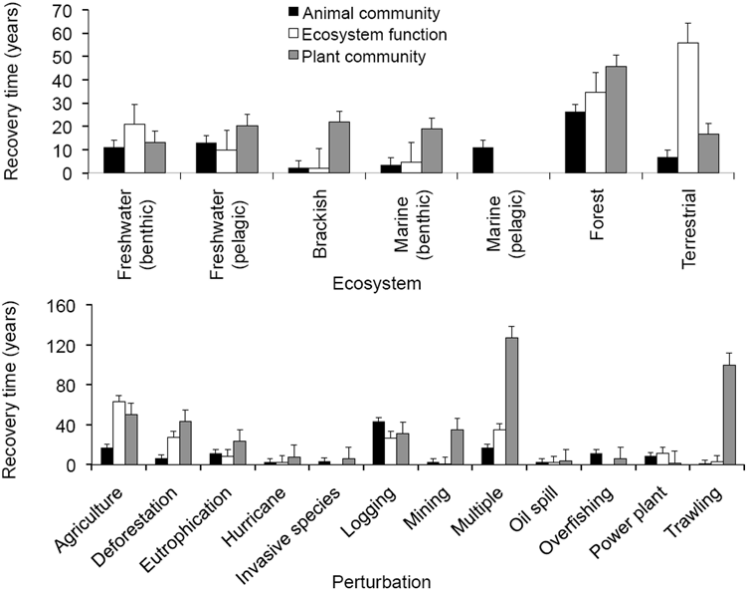
Average recovery times across ecosystems (top) and perturbation type (bottom). Variables are separated by animal community (black), ecosystem function (white) and plant community (gray) types. Bars represent mean±one standard error.

We found no discernable trend between community-level variables and ecosystem-level variables (Fig. 2). Because some ecosystems and perturbation types operate on different spatial and temporal scales, we looked at each variable type by each perturbation and ecosystem type separately and still found no patterns. Although theory indicates that different variable categories should respond on different timescales, we show there is no discernable pattern in the length of recovery for different variable types. This may be a result of the extraordinary number of variables measured amongst the studies (94 different variables).

We extracted data from those studies that provided pre-perturbation or initial conditions to calculate the magnitude of the perturbation. Perturbations could lead to an increase or a decrease in response variables relative to initial conditions. For example, species diversity could increase or decrease with a perturbation. We calculated perturbation magnitude as the percentage deviation from initial conditions (D) with the formula:
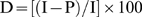
where P derives from quantitative measures of variables reported in the first time step following a perturbation and I indicates conditions prior to the perturbation for that datum. This index quantifies the potential for positive or negative directional change in a variable following a perturbation as alluded to above. We assessed whether or not there were systematic differences between positive and negative directional change. Having found none, we plotted all deviations as absolute values to facilitate comparison on a single graphical quadrant.

For each ecosystem and perturbation category, we compared index values of I_r_ and I_nr_ with paired t-tests after validating that the data were normally distributed. We used Analysis of Variance (ANOVA) with post-hoc Tukey tests to compare differences in average recovery times across ecosystem and perturbation categories, respectively. We tested for relationships between percentage deviation and recovery times using linear and non-linear regression. Values are significant at α = 0.05. Systat 10.2 was used to calculate all statistics.

## Results

**Figure 3:**
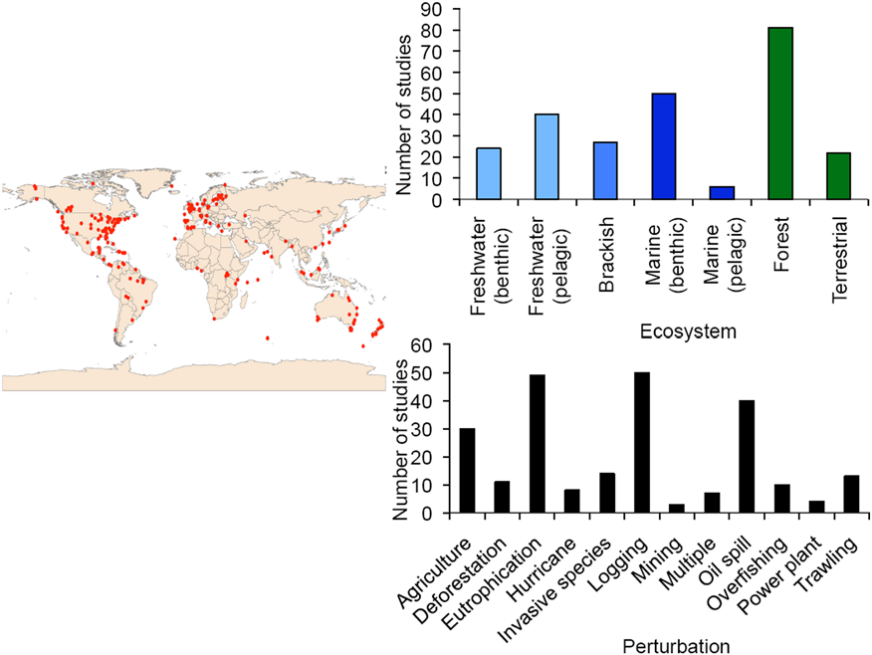
Geographic locations of the 240 studies used in the synthesis of ecosystem recovery (left). The number of studies in each ecosystem type (top right) and perturbation type (bottom right). The synthesis shows a high representation of studies across various biomes throughout the globe. Some ecosystem and disturbance types were more highly represented than others in the literature, as indicated by both graphs on the right. Colors represent the spectrum of aquatic to terrestrial ecosystems.

**Figure 4:**
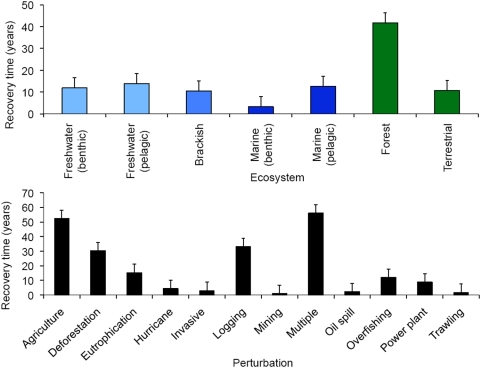
Average recovery times by ecosystem type (top) and perturbation type (bottom). Forests took longest to recover, whereas aquatic systems required less recovery time than terrestrial systems. Ecosystems took the longest to recover from agriculture, logging, and multiple stressors. Bars represent mean±one standard error. Colors represent the spectrum of aquatic to terrestrial ecosystems.

Our data set has broad global coverage of seven different aquatic and terrestrial ecosystem types (Fig. 3) and addresses recovery from major anthropogenic perturbations that these systems face [1]: agriculture, deforestation, eutrophication, invasive species, logging, mining, oil spill, overfishing, power plant, trawling, and interactions of those perturbations (multiple perturbations). We also compared these recovery times with those for major natural disturbances (hurricanes/cyclones). Our evidence does not support gloomy predictions [3], [15], but rather shows that there may be much hope to restore even heavily degraded ecosystems. Even more surprising, recovery can be much faster than the centuries and millennia speculated previously (Fig. 4).

We found 83 studies that demonstrated recovery for all variables, 90 studies reported a mixture of recovered and non-recovered variables, and 67 studies reported no recovery for any variable whatsoever. Among studies reporting recovery for any variable, the average recovery time was at most 42 years (for forest ecosystems) and typically much less (on the order of 10 years) when recovery was examined by ecosystem type (Fig. 4 top). When examined by perturbation type, the average recovery time was no more than 56 years (for systems undergoing multiple interacting perturbations) and typically was 20 years or less (Fig. 4 bottom). Most recovery from human disturbance was, however, slower than from natural causes (hurricanes/cyclones).

Because ecosystem variables (chemical and physical) and community variables (attributes of plant and animal species, including biodiversity) may operate on different time scales [16], [17], [18], [19], we further evaluated recovery for these two kinds of variable separately. We found no difference in return times between community and ecosystem variables (Fig. 2), suggesting that on average they operate on contemporary time scales.

Brackish, aquatic, and terrestrial grassland systems had statistically similar recovery times and collectively they recovered faster than terrestrial forest systems (ANOVA, p<0.001, d.f. = 6, 168, F = 7.217; followed by post-hoc Tukey pair-wise tests; Fig. 4). Recovery following agricultural activities and multiple perturbations was significantly slower than all other perturbation types (ANOVA, p<0.001, d.f. = 11, 163, F = 5.606, followed by post-hoc Tukey pair-wise tests: Fig. 4).

## Discussion

**Figure 5:**
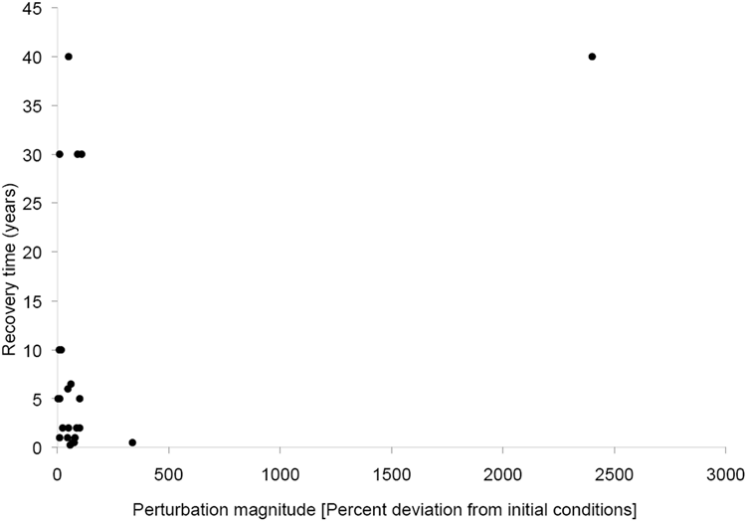
Relationship between deviation from initial conditions (perturbation magnitude) and the time taken for an ecosystem or community variable to recover. Data come from a small subset of the 240 studies that measured initial conditions and provided time series data to measure post disturbance levels. The significant relationship depends on the one outlier, indicating more information is needed on perturbations between 500–2000% deviation from initial conditions.

We found a significant positive relationship between perturbation magnitude and recovery time for variables that had fully recovered (Regression, r^2^ = 0.22, p<0.05, d.f. = 1, F = 6.3; Fig. 5). However, the significance was entirely determined by one strongly outlying point, implying perhaps that recovery may be independent of perturbation magnitude and instead idiosyncratic to the ecosystem type. For instance, turnover times for the longest living species and nutrient pools are shorter in aquatic than terrestrial systems [17], [18], [20], [21], [22], which may explain why aquatic systems trended towards shorter recovery times than terrestrial systems independently of disturbance magnitude. Ecosystems recovered more slowly following agriculture, deforestation and logging, but this is confounded by the fact that these disturbances exclusively impact terrestrial systems that generally recover more slowly than other systems. Regardless, in the balance, recovery can be quite rapid even from putatively very substantial perturbations (i.e. on the order of 100 to 300% change in variables; Fig. 5).

One potential pitfall of this assessment is the possibility that the systems were already in a disturbed state. Many ecosystems across the globe have faced large-scale perturbations including massive extinctions, abrupt species shifts, and changing disturbance regimes as a result of human activities. These perturbations, combined with lower-level sustained disturbances such as pollution, low-impact logging/farming, and climate shifts, could cause the baseline of many of the studies reviewed here to be far removed from a distant historical natural state. As such, the recovery of the variables in this review may mostly consist of variables that are easily measured by ecologists on contemporary timescales. However, it is noteworthy that historical reference sites are often not representative of ecosystem states that humans aspire to restore. As such, many restoration projects have moved away from the idea of restoring back to ‘natural’ or pre-human states and instead use contemporaneous reference systems as restoration targets [23].

Three explanations could account for lack of recovery in almost half of the systems and response variables. First, a particular study may not have been conducted over a long enough time scale to detect recovery. To assess this possibility, we compared the average recovery times for those ecosystems that we found to be fully recovered with the duration of those studies reporting that variables had not yet recovered. In 54% of the studies that reported unrecovered variables, the monitoring program did not likely run long enough to draw any definitive conclusion about recovery. Second, systems may have entrained into alternative states thereby precluding recovery. However, only 5% of the total studies (exclusively reported in the Resilience Alliance data set) conclusively reported that the ecosystems were irreversibly entrained into alternative states. Third, while some studies did rely on either a pre-perturbation or undisturbed control as an objective benchmark, this was not universally so. Of the 240 studies, only 20% used pre-perturbation data and 58% used undisturbed reference sites. Accordingly, the possibility existed that authors relied on an implicit and subjective definition of recovery for which conditions may or may not ever be realized based on their expert judgment. Nevertheless, our data set now provides a temporal benchmark for gauging recovery success.

Finally, there is a need for objective criteria to decide when a system has fully recovered. For deterministic systems, the plausible criterion is recovery to previous initial conditions. However, the stochasticity of natural systems means that they may never return to levels found in pre-perturbation conditions or that they may never have been in an initial equilibrium state. Rigorous quantitative methods exist to decide whether or not a variable has recovered in stochastic systems [24]. But, even so, our analysis shows that the prognosis for recovery will depend critically on the type of variable measured. We cannot at this time make any general claims about which variables best predict recovery. This creates a dilemma because in our analysis 94 different variables were measured (Fig. 2), all of which would be impossible to include in a single monitoring program.

The field of ecosystem conservation is at an important juncture [4]. We can either continue to chronicle ecosystem destruction [3], [5], [15] in hopes of spurring action to protect “natural” ecosystems by precluding humans from those areas. Or, we can recognize that humankind has and will continue to actively domesticate nature to meet its own needs [2], [4]. In the latter case, human agency will shape the nature and scale of impacts. Our results are not intended to give license to exploit ecosystems without regard to sustainability. But, with even the best sustainable practices unforeseen outcomes and damages can happen accidentally [1], [4]. The message of our paper is that recovery is possible and can be rapid for many ecosystems, giving much hope for humankind to transition to sustainable management of global ecosystems.

## Supporting Information

Table S1 Characteristics of studies covered in the synthesis of ecosystem recovery. Citations listed first are those that were used for data analysis.
(0.14 MB XLS)
